# Reliability and Validity of the Turkish Pain Behaviour Scale in Adults with Chronic Nonspecific Low Back Pain

**DOI:** 10.3390/jcm15135017

**Published:** 2026-06-27

**Authors:** Elif Esma Bayraktar, Irmak Çavuşoğlu, Yağmur İldeniz, Nuray Alaca

**Affiliations:** 1 Department of Physiotherapy and Rehabilitation, Faculty of Health Sciences, Acibadem Mehmet Ali Aydinlar University, Kerem Aydinlar Campus, Kayisdagi Street No. 32, Atasehir, 34752 Istanbul, Türkiye; yagmur.kucuk@acibadem.edu.tr (Y.İ.); nuray.alaca@acibadem.edu.tr (N.A.); 2 Department of Physiotherapy and Rehabilitation, Faculty of Health Sciences, Kırklareli University, 39100 Kırklareli, Türkiye; irmakcavusoglu@klu.edu.tr

**Keywords:** low back pain, pain measurement, psychometrics, reproducibility of results, observer variation, Pain Behaviour Scale

## Abstract

**Background/Objectives**: Pain behaviors observed during movement may complement self-reported and performance-based assessment in chronic nonspecific low back pain (NSLBP). The Pain Behaviour Scale (PaBS) quantifies clinician-observed pain behavior severity during functional tasks, but no Turkish version has been evaluated. This study translated and culturally adapted the PaBS into Turkish and examined its reliability, agreement, measurement error, and construct validity. **Methods**: This cross-cultural adaptation and psychometric validation study included 102 adults with chronic NSLBP. The PaBS was translated, back-translated, reviewed by an expert panel, and pilot tested. Participants completed clinical questionnaires and standardized physical performance tests. Two independent raters scored the PaBS at baseline; one rater repeated scoring after one week. Reliability was analyzed using ICCs. Measurement error, agreement, and construct validity were assessed using SEM, MDC95, percentage agreement, Cohen’s kappa, Bland–Altman analysis, and predefined correlation hypotheses. **Results**: Total-score interrater reliability was excellent (ICC = 0.95), and intrarater reliability was high (ICC = 0.96), although the latter should be interpreted cautiously because participant status changed between sessions. MDC95 values were 2.05 and 1.85. Individual behavior agreement ranged from 81.4% to 100.0%, but item-level findings should be supplementary. All construct-validity correlations were in the expected direction; however, most were stronger than predefined expectations, and only 1 of 13 hypotheses met both direction and magnitude. **Conclusions**: The Turkish PaBS appears reliable for assessing observed pain behavior severity during functional movement in adults with chronic NSLBP. Construct-validity findings should be considered preliminary because stronger-than-expected correlations may reflect construct overlap with disability, fear-avoidance, and physical performance.

## 1. Introduction

Chronic nonspecific low back pain (NSLBP) is a highly prevalent musculoskeletal condition associated with persistent pain, functional limitation, reduced work participation, and impaired quality of life [1,2]. Contemporary evidence emphasizes that chronic low back pain (LBP) should be understood as a multidimensional condition influenced by biological, psychological, behavioral, and social factors [1,3]. Therefore, assessment should not rely solely on pain intensity or disability scores but should also consider how pain is expressed during functional movement.

Pain behaviors represent an observable behavioral dimension of the pain experience. These behaviors may include grimacing, sighing, breath-holding, guarding, rubbing, altered movement patterns, and antalgic gait [3,4]. However, pain behaviors should not be interpreted as direct proxies for pain severity, disability, or symptom exaggeration. Observable behaviors during movement may reflect pain, fear-related movement behavior, learned protective strategies, visible movement limitation, or task-specific functional difficulty. Accordingly, pain behaviors may provide complementary clinical information when interpreted alongside self-reported symptoms, disability measures, and physical performance tests [4,5].

Despite their potential clinical relevance, pain behaviors are not routinely assessed using standardized, feasible, and psychometrically supported tools. Earlier observational approaches have often focused on the presence or frequency of pain behaviors, relied on videotaped assessment, or evaluated patients during nonfunctional examination procedures [3,4]. Frequency-based observation alone may be insufficient because it does not distinguish between individuals who display similar numbers of behaviors but differ in the severity of their behavioral pain expression [3]. A practical observational measure that evaluates both the presence and severity of pain behaviors during functional movement may therefore add clinically meaningful information to conventional self-report and performance-based assessment.

The Pain Behaviour Scale (PaBS) was developed as a clinician-rated observational measure to assess pain behavior severity during functional movement in individuals with chronic LBP [3]. Its performance-based structure is consistent with current approaches that emphasize multidimensional assessment and the integration of functional tasks in the evaluation of lumbar disorders [6]. Previous studies have reported favorable reliability and clinically meaningful associations with pain intensity, disability, and physical performance [3,7]. However, existing PaBS studies have used relatively limited sample sizes, and further psychometric evaluation is needed across different languages, cultures, and clinical populations. In addition, recent evidence on direct observation of pain behaviors highlights that observational methods may be clinically useful but require careful psychometric evaluation because pain behaviors are context-dependent and may overlap with broader functional limitations [4].

Before implementation in Turkish-speaking clinical and research settings, cross-cultural adaptation and psychometric evaluation of the PaBS are required to ensure conceptual equivalence, clinical interpretability, and measurement reliability [8,9]. To date, the Turkish version of the PaBS has not been psychometrically evaluated in individuals with chronic NSLBP. Given the potential overlap between observed pain behavior, disability, fear-avoidance, and physical performance, validation of the Turkish PaBS requires not only reliability and agreement testing but also cautious construct-validity evaluation based on predefined hypotheses.

Therefore, the aim of this study was to translate and culturally adapt the PaBS into Turkish and to examine its interrater reliability, intrarater reliability, agreement, measurement error, and construct validity in adults with chronic NSLBP.

## 2. Materials and Methods

### 2.1. Study Design and Ethics

This study was designed as a cross-cultural adaptation and psychometric validation study of the Turkish version of the PaBS in adults with chronic NSLBP. The design and reporting of measurement properties were guided by COSMIN terminology and recommendations, and the reliability and agreement components were structured according to the Guidelines for Reporting Reliability and Agreement Studies (GRRAS) [9,10,11]. Ethical approval was obtained from the Acibadem Mehmet Ali Aydinlar University Medical Research Evaluation Committee (ATADEK) on 6 October 2023, with decision number 2023-15/518. All participants provided written informed consent before enrollment, and the study was conducted in accordance with the Declaration of Helsinki.

### 2.2. Translation and Cross-Cultural Adaptation

Permission to translate and culturally adapt the PaBS into Turkish was obtained from the original developer. The translation process followed established cross-cultural adaptation recommendations for health measurement instruments [8]. Three independent bilingual researchers/physiotherapists produced the forward translations, which were synthesized by the research team. The synthesized version was back-translated into English by an independent bilingual translator who was blinded to the original scale.

The synthesized Turkish version and the back-translation were reviewed by an expert panel of four members. The panel included physiotherapists with at least 10 years of clinical and/or academic experience in musculoskeletal rehabilitation and pain assessment, as well as researchers experienced in cross-cultural adaptation of clinical outcome measures. The independent blinded back-translator also contributed to the language review during the back-translation and reconciliation process. The panel evaluated the Turkish version for semantic, idiomatic, conceptual, clinical, and procedural equivalence. Any wording or scoring-related ambiguities were discussed within the research team and clarified, when necessary, through correspondence with the original developers.

### 2.3. Pilot Testing

The prefinal Turkish version was pilot-tested in 20 individuals with chronic NSLBP who met the same age range and general eligibility criteria as the main study sample. Participants and raters were asked to report unclear instructions or procedural difficulties. No major cultural incompatibility was identified, as the PaBS is based on clinician observation during standardized clinical field tests. Minor procedural clarifications regarding instructions, rater positioning, and scoring were finalized before the main study. Pilot data were not included in the psychometric analyses.

### 2.4. Participants and Setting

Participants were recruited from the physical therapy and rehabilitation outpatient clinics of private hospitals and a university-based physiotherapy and rehabilitation education and research laboratory. Eligible participants were adults aged 18–55 years with chronic NSLBP lasting longer than 3 months. Participants were required to have sufficient cognitive and language ability to understand instructions, complete questionnaires, and provide informed consent.

Exclusion criteria were clinical features suggesting serious spinal pathology, including malignancy, infection, fracture, or cauda equina syndrome; spinal stenosis; signs of radiculopathy, including radiating leg pain, weakness, numbness, or tingling; previous fracture or surgery in the relevant spinal region; history of rheumatic disease; and pregnancy [3,12]. A minimum sample size of 100 participants was planned based on COSMIN-based methodological quality criteria for measurement-property studies, in which sample sizes of 100 or more are considered excellent [13]. This sample size was considered appropriate for the main total-score analyses, including estimation of interrater and intrarater ICCs, measurement error, and correlation-based testing of the predefined construct-validity hypotheses. However, because agreement estimates for individual pain behaviors depend on the prevalence and marginal distribution of each behavior, kappa coefficients for infrequently observed behaviors were interpreted cautiously.

### 2.5. Procedures

Participants attended two testing sessions separated by one week before the initiation of treatment, in order to minimize recall bias while reducing the likelihood of clinical change. At the first session, demographic and clinical characteristics were recorded, and participants completed self-report measures of pain intensity, pain characteristics, disability, and fear-avoidance beliefs. Participants subsequently performed the standardized physical performance tests, during which two independent physiotherapists with 13 and 18 years of experience in musculoskeletal pain assessment evaluated pain behaviors using the PaBS.

Standardized verbal instructions were provided before each physical performance test, and the corresponding time or distance values were recorded. During each test, two raters independently and simultaneously assessed the presence and severity of pain behaviors using PaBS while remaining blinded to each other’s scores. For intrarater reliability, the same rater repeated the PaBS assessment at the second session and was blinded to the previous ratings. Because all participants had chronic NSLBP, blinding of the raters to participants’ clinical condition was not possible. Before data collection, both raters completed a standardization meeting covering the Turkish PaBS instructions, behavioral definitions, 0–3 severity scoring, rater positioning, and standardized administration of the physical performance tests. Scoring-related ambiguities were clarified through team discussion and, when required, correspondence with the original developers.

### 2.6. Measures

#### 2.6.1. Pain Behaviour Scale

PaBS is a clinician-rated observational scale developed to assess the presence and severity of pain behaviors during standardized physical performance tests in individuals with chronic LBP [3]. The observed behaviors include sighing, breath-holding, grimacing, guarding, rubbing, and antalgic gait. For each physical performance test, the presence or absence of each behavior is recorded, and the overall severity of observed pain behavior is scored on a 4-point scale from 0 to 3. The total severity score ranges from 0 to 15, with higher scores indicating greater severity of observed pain behaviors.

#### 2.6.2. Physical Performance Tests

PaBS was administered during five standardized physical performance tests based on the functional test battery described by Simmonds et al. [14]: repeated trunk flexion, repeated sit-to-stand, timed up and go, loaded reach, and 50-foot walk. Time-based tests were recorded in seconds, and loaded reach was recorded in centimeters.

#### 2.6.3. Self-Report Measures

Pain intensity and pain characteristics were assessed using the Short-Form McGill Pain Questionnaire (SF-MPQ), which includes sensory and affective descriptors as well as a visual analog scale (MPQ-VAS) component for pain intensity [15]. Disability related to low back pain was assessed using the Oswestry Disability Index (ODI); the Turkish version has demonstrated reliability and validity in patients with LBP [16]. Fear-avoidance beliefs were assessed using the Fear-Avoidance Beliefs Questionnaire (FABQ); the Turkish version has demonstrated reliability and validity in patients with LBP [17].

#### 2.6.4. Reliability and Agreement

Interrater reliability of the Turkish PaBS was evaluated using scores recorded independently and simultaneously by two raters during the first testing session. Intrarater reliability was evaluated using scores recorded by the same rater across the two testing sessions. Test–retest reliability of the physical performance test components was evaluated using the time or distance values recorded across the two sessions.

Reliability was evaluated for the PaBS total score and task-specific PaBS scores. Agreement was evaluated for the presence or absence of individual pain behaviors. Internal consistency was not assessed because the PaBS is a multidimensional observational measure composed of distinct pain behavior indicators and task-specific severity ratings rather than a unidimensional scale with interchangeable items. Therefore, internal consistency coefficients such as Cronbach’s alpha were not considered appropriate for this instrument.

#### 2.6.5. Construct Validity

Construct validity was examined using hypothesis testing in accordance with COSMIN principles [9,10]. Higher Turkish PaBS scores were expected to reflect greater pain-related clinical burden and poorer physical performance. A priori, fair-to-moderate positive correlations were expected with pain intensity, disability, fear-avoidance beliefs, and time-based physical performance measures, whereas fair-to-moderate negative correlations were expected with loaded reach distance. Construct validity was evaluated according to both the expected direction and magnitude of these predefined hypotheses. Correlations substantially stronger than the predefined range were interpreted cautiously as potentially indicating construct overlap rather than unequivocal evidence of validity.

#### 2.6.6. Statistical Analysis

Statistical analyses were performed using IBM SPSS Statistics, version 21.0. Descriptive statistics were used to summarize demographic and clinical characteristics. Continuous variables were reported as means and standard deviations or medians and interquartile ranges, according to data distribution, whereas categorical variables were reported as frequencies and percentages. Data distribution was assessed using visual inspection and normality tests.

Participant stability between the two testing sessions was examined by comparing pain intensity, disability, PaBS total scores, and physical performance test scores using paired-samples *t* tests or Wilcoxon signed-rank tests, as appropriate [3]. Interrater reliability of the PaBS total and task-specific severity scores was analyzed using a two-way random-effects, absolute-agreement, single-measure intraclass correlation coefficient [ICC(2, 1)]. Intrarater reliability was analyzed using a two-way mixed-effects, absolute-agreement, single-measure intraclass correlation coefficient [ICC(3, 1)] [18,19]. Test–retest reliability of the physical performance test components was analyzed using ICC(1, 1), consistent with the original PaBS study [3]. In accordance with COSMIN recommendations, ICC values greater than 0.70 were considered sufficient for reliability. Conventional descriptive categories were used as supplementary interpretation, with ICC values interpreted as poor (<0.50), moderate (0.50–0.75), good (0.75–0.90), or excellent (>0.90) [18].

Measurement error was quantified using the standard error of measurement, calculated as SDpooled × √(1 − ICC) [20]. The minimal detectable change at the 95% confidence level was calculated as SEM × 1.96 × √2 [20,21]. Agreement for the presence or absence of individual pain behaviors was examined using Cohen’s kappa coefficients and percentage agreement [22,23]. Because several pain behaviors were expected to occur infrequently, item-level kappa analyses were interpreted as exploratory and supplementary to the total-score reliability analyses. Absolute agreement for PaBS total scores was examined using Bland–Altman analysis and 95% limits of agreement [24].

Construct validity was assessed using Pearson or Spearman correlation coefficients according to data distribution. Correlation coefficients below 0.25 were interpreted as weak, 0.25–0.50 as fair, 0.50–0.75 as moderate, and above 0.75 as strong. Construct validity was considered adequate when at least 75% of the predefined hypotheses were confirmed with respect to both direction and magnitude [9,10,13]. Statistical significance was set at *p* < 0.05; however, construct validity was interpreted primarily according to hypothesis confirmation rather than statistical significance alone.

## 3. Results

### 3.1. Participant Characteristics

**Figure 1:**
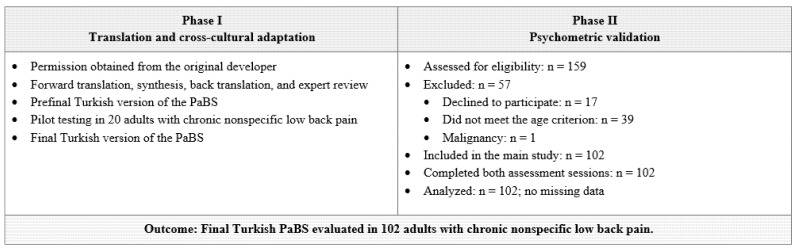
Flow diagram of the Turkish Pain Behaviour Scale adaptation and validation process. The figure summarizes the translation, cross-cultural adaptation, pilot testing, and main psychometric validation process of the Turkish version of the Pain Behaviour Scale (PaBS). Detailed procedural and statistical information is provided in Section 2.

A total of 102 individuals with chronic NSLBP were included in the final analysis, exceeding the planned minimum sample size of 100 participants (Figure 1). The mean age of the participants was 39.0 ± 9.5 years, and the mean BMI was 25.9 ± 1.5 kg/m^2^. Sixty-three participants were women, representing 61.8% of the sample. The mean duration of LBP was 12.5 ± 6.8 months. At baseline, the mean MPQ-VAS pain intensity score was 4.8 ± 0.6, the mean SF-MPQ total score was 11.9 ± 2.5, the mean ODI score was 28.0 ± 6.4%, and the mean FABQ total score was 37.8 ± 5.3. Baseline demographic and clinical characteristics are presented in Table 1.

### 3.2. Between-Session Changes in Clinical and Physical Performance Measures

Clinical outcomes, physical performance measures, and PaBS total scores across the two testing sessions are presented in Table 2. Statistically significant between-session differences were observed for SF-MPQ total score, MPQ-VAS pain intensity, ODI score, PaBS total score, and all physical performance measures, whereas FABQ total score did not differ significantly between sessions.

The SF-MPQ total score decreased from 11.9 ± 2.5 to 10.7 ± 2.4, MPQ-VAS pain intensity from 4.8 ± 0.6 to 4.6 ± 0.7, ODI score from 28.0 ± 6.4% to 26.6 ± 5.7%, and PaBS total score from 3.9 ± 3.5 to 3.2 ± 2.9. Among the physical performance measures, repeated trunk flexion, repeated sit-to-stand, Timed Up and Go, and 50-foot walk completion times decreased, whereas loaded reach distance increased.

These between-session differences indicate that the clinical and performance status of the sample was not identical across the two testing sessions.

### 3.3. Reliability and Measurement Error of the Turkish PaBS

Reliability and measurement error results for the Turkish PaBS are presented in Table 3. Interrater ICC values for task-specific PaBS scores ranged from 0.76 to 0.97. The highest interrater ICC was observed for loaded reach, and the lowest interrater ICC was observed for repeated sit-to-stand. The interrater ICC for the total PaBS score was 0.95, with a 95% confidence interval of 0.91 to 0.98. The SEM for the total PaBS score was 0.74, and the MDC95 was 2.05 points.

Intrarater ICC values for task-specific PaBS scores ranged from 0.82 to 0.96. The intrarater ICC for the total PaBS score was 0.96, with a 95% confidence interval of 0.94 to 0.97. The SEM for the total PaBS score was 0.67, and the MDC95 was 1.85 points.

Interrater reliability was assessed by two raters within the same testing session, whereas intrarater reliability was assessed by the same rater across the two testing sessions.

### 3.4. Test–Retest Reliability of Physical Performance Tests

Test–retest reliability coefficients for the physical performance tests are shown in Table 3. ICC values ranged from 0.82 to 0.99. The ICC values were 0.96 for repeated trunk flexion, 0.98 for repeated sit-to-stand, 0.82 for the Timed Up and Go test, 0.99 for loaded reach, and 0.98 for the 50-foot walk test. SEM values ranged from 0.45 cm for loaded reach to 2.44 s for repeated trunk flexion. MDC95 values ranged from 1.25 cm for loaded reach to 6.76 s for repeated trunk flexion.

### 3.5. Interrater Agreement for Individual Pain Behaviors

Interrater agreement for the presence of individual pain behaviors during the physical performance tests is presented in Table 4. Percentage agreement ranged from 81.4% to 100.0% across behaviors and tasks.

During repeated trunk flexion, Cohen’s κ values ranged from 0.41 for guarding to 1.00 for grimacing. During repeated sit-to-stand, κ values ranged from 0.42 for sighing to 0.70 for guarding. During the Timed Up and Go test, κ values ranged from 0.00 for breath-holding to 0.65 for antalgic gait; κ could not be calculated for grimacing because neither rater recorded the behavior.

During loaded reach, κ values ranged from 0.00 for rubbing to 1.00 for breath-holding and guarding. During the 50-foot walk test, κ values ranged from 0.09 for guarding to 0.82 for breath-holding. Antalgic gait was assessed only during walking-related tasks and showed κ values of 0.65 for the Timed Up and Go test and 0.78 for the 50-foot walk test.

Across behaviors and tasks, κ coefficients showed greater variability than percentage agreement values. Low or zero κ coefficients were observed for some infrequently recorded or task-dependent behaviors, including breath-holding during the Timed Up and Go test, rubbing during loaded reach, and guarding during the 50-foot walk test.

### 3.6. Construct Validity

Correlations between Turkish PaBS total scores and clinical and physical performance measures are presented in Table 5. The PaBS total score showed positive correlations with all self-reported clinical measures, including SF-MPQ affective score (ρ = 0.82), SF-MPQ sensory score (ρ = 0.84), SF-MPQ total score (ρ = 0.85), MPQ-VAS pain intensity (ρ = 0.86), ODI disability score (ρ = 0.85), FABQ physical activity (ρ = 0.93), FABQ work (ρ = 0.84), and FABQ total score (ρ = 0.90). For physical performance measures, the PaBS total score was positively correlated with repeated trunk flexion (ρ = 0.93), repeated sit-to-stand (ρ = 0.93), Timed Up and Go (ρ = 0.93), and 50-foot walk time (ρ = 0.67), and negatively correlated with loaded reach distance (ρ = −0.93). All correlations were statistically significant at *p* < 0.001 and were in the hypothesized direction.

When the predefined hypotheses were evaluated according to both direction and magnitude, 1 of 13 hypotheses met the expected fair-to-moderate correlation range. Accordingly, the COSMIN-based criterion requiring confirmation of at least 75% of predefined hypotheses with respect to both direction and magnitude was not met. Correlations exceeding the predefined range were observed particularly for fear-avoidance measures and physical performance tests.

### 3.7. Bland–Altman Agreement

Bland–Altman agreement results for PaBS total scores are presented in Table 6. For interrater agreement, the mean difference between Rater 2 and Rater 1 during the first testing session was −0.03 points, with 95% limits of agreement from −2.08 to 2.02. For intrarater agreement, the mean difference between the second and first testing sessions for Rater 1 was −0.74 points, with 95% limits of agreement from −2.58 to 1.11.

## 4. Discussion

This study provides the first evidence on the cross-cultural adaptation and psychometric properties of the Turkish version of the PaBS in adults with chronic NSLBP. The main findings indicate strong same-session interrater reliability and acceptable measurement error for the PaBS total severity score. The across-session intrarater ICC was also high; however, this estimate should be interpreted cautiously because statistically significant between-session changes were observed in pain intensity, disability, PaBS total score, and physical performance measures. Construct-validity testing showed that all predefined correlations were in the expected direction, but most exceeded the predefined fair-to-moderate magnitude range. Therefore, the findings support the Turkish PaBS primarily as a reliable observational measure, whereas construct validity should be regarded as preliminary and requiring further investigation.

The reliability findings suggest that the Turkish PaBS total score can be scored consistently when standardized administration and rater procedures are used. This is consistent with the original PaBS study, which reported excellent reliability in individuals with chronic LBP [3]. In the present study, the interrater reliability results provide the most direct evidence of reproducibility because both raters assessed participants during the same testing session. The negligible mean interrater difference observed in the Bland–Altman analysis also indicates limited systematic difference between raters. In contrast, the intrarater reliability and across-session Bland–Altman findings require a more cautious interpretation. Because participants showed statistically significant changes over the one-week interval, these estimates may reflect both rater consistency and short-term participant variability, including symptom fluctuation, familiarization with the tasks, learning effects, or habituation. Although the mean between-session change in PaBS total score was below the intrarater MDC95, the absence of complete participant stability limits the interpretation of intrarater reliability as a pure estimate of rating consistency.

The SEM and MDC95 values provide useful information for interpreting measurement error of the Turkish PaBS total score. The MDC95 values of approximately two points suggest the minimum change required to exceed expected measurement error at the individual level. However, these values should not be interpreted as minimal clinically important differences, because anchor-based responsiveness and patient-centered change criteria were not examined in this study [18,20]. Future research should therefore investigate responsiveness and clinically meaningful change thresholds before the Turkish PaBS is used to evaluate treatment effects.

The findings further support the total PaBS severity score as the primary, most stable, and clinically interpretable outcome of the scale. Although percentage agreement for individual pain behaviors was generally high, kappa coefficients varied across behaviors and tasks, with low or zero values observed for some infrequently recorded behaviors. These item-level findings should be interpreted cautiously, as kappa statistics are sensitive to low prevalence and imbalanced marginal distributions [23,25]. In addition, behaviors such as breath-holding, rubbing, and guarding may be brief, intermittent, or task-dependent, which may reduce kappa values despite high percentage agreement. Therefore, agreement results for individual pain behaviors should be regarded as supplementary descriptive information rather than independent clinical endpoints. This interpretation is consistent with previous literature indicating that direct observation of pain behavior is clinically relevant but methodologically complex because pain behaviors are context-dependent, intermittent, and variably expressed across functional tasks [4].

The construct-validity findings should be interpreted cautiously. Higher PaBS scores were associated with greater pain intensity, disability, fear-avoidance beliefs, slower time-based performance, and shorter loaded reach distance, consistent with evidence that chronic LBP-related disability reflects interactions among pain, psychological factors, movement behavior, and functional capacity [1,12,26]. However, fair-to-moderate correlations were predefined because the PaBS was intended to assess an observational construct related to, but not interchangeable with, self-reported clinical burden and physical performance. Although all correlations were statistically significant and in the expected direction, only 1 of 13 hypotheses met both the expected direction and magnitude criteria; therefore, the COSMIN criterion requiring confirmation of at least 75% of predefined hypotheses was not met [27]. The stronger-than-expected correlations, particularly with FABQ physical activity and time-based performance tests, may reflect visible movement limitation, slowed task execution, fear-related movement behavior, or shared functional-task context rather than definitive evidence of construct validity. Recent work on pain-related fear and lumbar movement behavior similarly supports interpreting movement-based behavioral findings in relation to task demands and psychological context [28,29]. Thus, these findings should be considered preliminary correlational evidence rather than adequate confirmation that the PaBS captures a fully distinct observational construct.

Clinically, the Turkish PaBS may help standardize the observation of pain behaviors that clinicians often recognize informally during physical assessment, including guarding, breath-holding, grimacing, rubbing, sighing, and antalgic gait. Its value lies in adding a structured behavioral observation component to the multidimensional assessment of chronic NSLBP. Pain intensity, disability questionnaires, fear-avoidance measures, and physical performance tests each capture different but related aspects of the pain experience. The PaBS may therefore be particularly useful when clinicians need to compare self-reported symptoms, observed movement behavior, and functional performance. However, PaBS scores should not be used as direct proxies for pain severity, disability, symptom credibility, or exaggeration. Observed pain behavior should instead be interpreted as one component of the broader biopsychosocial presentation of chronic LBP [1,4,12,26].

This study has several strengths. The Turkish PaBS was developed through a structured translation and cross-cultural adaptation process, including expert review and pilot testing. The sample size was appropriate for the main total-score reliability and construct-validity analyses, and the study included two independent raters, standardized functional performance tests, SEM and MDC95 estimates, Bland–Altman analysis, and COSMIN-informed hypothesis testing. These features strengthen the methodological basis for using the Turkish PaBS total score in adults with chronic NSLBP.

Several limitations should also be acknowledged. First, complete participant stability across the one-week interval cannot be assumed because statistically significant between-session changes were observed in PaBS total score, pain intensity, disability, and physical performance measures. Therefore, across-session intrarater reliability and Bland–Altman findings may reflect both rating consistency and short-term participant variability. Second, construct validity was examined only through correlation-based hypothesis testing. Known-groups validity, discriminant validity, predictive validity, responsiveness, and comparisons with another observational pain-behavior measure were not evaluated. Third, most correlations exceeded the predefined fair-to-moderate range, which may reflect construct overlap or shared task context rather than independent evidence of a distinct construct. Fourth, although the overall sample size was adequate for the main analyses, the stability of item-level kappa estimates may have been limited for behaviors with low prevalence or imbalanced marginal distributions. Fifth, participants were recruited using a non-probabilistic sampling strategy from outpatient clinics and a university-based rehabilitation laboratory, which may limit generalizability. Finally, the sample consisted of adults with chronic NSLBP without radiculopathy or serious spinal pathology; therefore, the findings should be generalized cautiously to older adults, postsurgical populations, individuals with radicular symptoms, or patients with more severe disability.

Future studies should further evaluate the Turkish PaBS using broader COSMIN measurement-property frameworks. In particular, discriminant validity, known-groups validity, responsiveness, minimal clinically important difference, and clinical interpretability should be examined. Studies comparing the PaBS with other observational pain-behavior measures or testing its performance across different clinical subgroups would help clarify whether the scale captures a distinct observational construct or primarily reflects broader pain-related functional limitation.

## 5. Conclusions

In conclusion, the Turkish version of the PaBS demonstrated excellent interrater reliability and acceptable measurement error for the total severity score in adults with chronic nonspecific low back pain. Although intrarater reliability was high, this finding should be interpreted in light of the observed between-session changes, indicating that complete participant stability over the one-week interval cannot be fully assumed. The total PaBS severity score appears to be the primary, most stable, and clinically interpretable outcome of the scale. In contrast, agreement findings for individual pain behaviors should be regarded as supplementary descriptive information and interpreted cautiously, particularly for infrequently observed or task-dependent behaviors.

The construct-validity findings suggest preliminary, potentially clinically relevant associations between Turkish PaBS scores and clinical and performance-based measures within the context of functional movement assessment. However, the stronger-than-expected associations with disability, fear-avoidance beliefs, and physical performance measures indicate that observed pain behavior may partly reflect shared task context, visible movement limitation, slowed task execution, or fear-related movement behavior. Therefore, the Turkish PaBS may serve as a structured observational component within multidimensional assessment in chronic nonspecific low back pain, particularly when interpreted alongside self-reported symptoms, disability, fear-avoidance beliefs, and physical performance measures. Further studies are warranted to examine its discriminant validity, known-groups validity, responsiveness, and interpretability of change scores in different clinical samples and settings.

## Figures and Tables

**Table 1:** Baseline demographic and clinical characteristics of participants.

Characteristic	Value
**Demographic characteristics**	
Age, years	39.0 (9.5)
**Sex**	
Female	63 (61.8%)
Male	39 (38.2%)
Body mass index, kg/m^2^	25.9 (1.5)
**Marital status**	
Married	65 (63.7%)
Unmarried	37 (36.3%)
**Smoking status**	
Smoker	19 (18.6%)
Nonsmoker	83 (81.4%)
**Education**	
No formal education	3 (2.9%)
High school	26 (25.5%)
University degree	66 (64.7%)
Postgraduate degree	7 (6.9%)
**Occupation**	
Office worker	23 (22.5%)
Professional worker	48 (47.1%)
Other	31 (30.4%)
**Clinical characteristics**	
Pain duration, months	12.5 (6.8)
MPQ-VAS pain intensity	4.8 (0.6)
SF-MPQ affective score	4.0 (1.1)
SF-MPQ sensory score	8.0 (1.4)
SF-MPQ total score	11.9 (2.5)
ODI disability, %	28.0 (6.4%)
FABQ physical activity	15.2 (2.3)
FABQ work	22.6 (3.1)
FABQ total	37.8 (5.3)

Values are presented as mean (SD) or *n* (%). Clinical measures refer to the first testing session. MPQ-VAS, McGill Pain Questionnaire Visual Analog Scale; SF-MPQ, Short-Form McGill Pain Questionnaire; ODI, Oswestry Disability Index; FABQ, Fear-Avoidance Beliefs Questionnaire.

**Table 2:** Between-session changes in clinical and physical performance measures.

Measure	First Session, Mean (SD)	Second Session, Mean (SD)	Mean Difference (95% CI)	*p* Value
**Clinical measures**				
SF-MPQ total score	11.9 (2.5)	10.7 (2.4)	−1.25 (−1.41 to −1.10)	<0.001
MPQ-VAS pain intensity	4.8 (0.6)	4.6 (0.7)	−0.20 (−0.23 to −0.18)	<0.001
ODI disability, %	28.0 (6.4)	26.6 (5.7)	−1.32 (−1.56 to −1.09)	<0.001
FABQ total score	37.8 (5.3)	37.8 (4.8)	−0.01 (−0.25 to 0.23)	0.938
PaBS total score	3.9 (3.5)	3.2 (2.9)	−0.74 (−0.92 to −0.55)	<0.001
**Physical performance measures**				
Repeated trunk flexion, s	31.6 (13.2)	28.2 (12.8)	−3.39 (−3.54 to −3.24)	<0.001
Repeated sit-to-stand, s	18.8 (6.9)	17.7 (6.2)	−1.12 (−1.28 to −0.96)	<0.001
Timed Up and Go, s	11.3 (3.7)	10.1 (2.5)	−1.21 (−1.50 to −0.92)	<0.001
Loaded reach, cm	16.2 (4.1)	16.7 (4.1)	0.50 (0.42 to 0.57)	<0.001
50-foot walk, s	27.0 (4.5)	26.6 (4.3)	−0.43 (−0.58 to −0.28)	<0.001

Values are presented as mean (SD). Mean differences were calculated as second session minus first session; negative values indicate lower scores or shorter completion times at the second session. Between-session comparisons were performed using Wilcoxon signed-rank tests because paired differences were not normally distributed. SF-MPQ, Short-Form McGill Pain Questionnaire; MPQ-VAS, McGill Pain Questionnaire Visual Analog Scale; ODI, Oswestry Disability Index; FABQ, Fear-Avoidance Beliefs Questionnaire; PaBS, Pain Behaviour Scale.

**Table 3:** Reliability and measurement error of the Turkish Pain Behaviour Scale and physical performance tests.

Measure	ICC (95% CI)	SEM	MDC95
**A. Interrater reliability of PaBS scores**			
Repeated trunk flexion	0.93 (0.90 to 0.96)	0.30	0.82
Repeated sit-to-stand	0.76 (0.59 to 0.89)	0.40	1.12
Timed Up and Go	0.78 (0.64 to 0.86)	0.34	0.95
Loaded reach	0.97 (0.94 to 0.99)	0.16	0.43
50-foot walk	0.88 (0.78 to 0.94)	0.23	0.64
Total PaBS score	0.95 (0.91 to 0.98)	0.74	2.05
**B. Intrarater reliability of PaBS scores**			
Repeated trunk flexion	0.90 (0.87 to 0.93)	0.33	0.92
Repeated sit-to-stand	0.96 (0.92 to 0.99)	0.15	0.43
Timed Up and Go	0.87 (0.80 to 0.92)	0.25	0.69
Loaded reach	0.82 (0.75 to 0.88)	0.36	1.00
50-foot walk	0.82 (0.68 to 0.92)	0.26	0.72
Total PaBS score	0.96 (0.94 to 0.97)	0.67	1.85
**C. Test–retest reliability of physical performance tests**			
Repeated trunk flexion, s	0.96 (0.95 to 0.98)	2.44	6.76
Repeated sit-to-stand, s	0.98 (0.97 to 0.98)	0.97	2.70
Timed Up and Go, s	0.82 (0.75 to 0.88)	1.32	3.65
Loaded reach, cm	0.99 (0.98 to 0.99)	0.45	1.25
50-foot walk, s	0.98 (0.97 to 0.99)	0.61	1.69

Interrater reliability of PaBS scores was calculated between the two raters during the first testing session. Intrarater reliability of PaBS scores was calculated for Rater 1 across the two testing sessions. Test–retest reliability of physical performance tests was calculated using time or distance values recorded across the two testing sessions. ICC, intraclass correlation coefficient; SEM, standard error of measurement; MDC95, minimal detectable change at the 95% confidence level; PaBS, Pain Behaviour Scale.

**Table 4:** Interrater agreement for the presence of pain behaviors during physical performance tests.

Physical Performance Test	Pain Behavior	R1 Yes, *n* (%)	R2 Yes, *n* (%)	Agreement, %	Cohen’s κ
**Repeated trunk flexion**	Sighing	60 (58.8)	66 (64.7)	94.1	0.88
	Breath-holding	33 (32.4)	39 (38.2)	94.1	0.87
	Grimacing	29 (28.4)	29 (28.4)	100.0	1.00
	Guarding	24 (23.5)	15 (14.7)	81.4	0.41
	Rubbing	24 (23.5)	25 (24.5)	97.1	0.92
**Repeated sit-to-stand**	Sighing	14 (13.7)	21 (20.6)	83.3	0.42
	Breath-holding	29 (28.4)	20 (19.6)	87.3	0.65
	Grimacing	5 (4.9)	6 (5.9)	95.1	0.52
	Guarding	18 (17.6)	14 (13.7)	92.2	0.70
	Rubbing	5 (4.9)	6 (5.9)	95.1	0.52
**Timed Up and Go**	Sighing	21 (20.6)	18 (17.6)	87.3	0.59
	Breath-holding	5 (4.9)	0 (0.0)	95.1	0.00
	Grimacing	0 (0.0)	0 (0.0)	100.0	NC
	Guarding	5 (4.9)	5 (4.9)	94.1	0.37
	Rubbing	10 (9.8)	9 (8.8)	93.1	0.59
	Antalgic gait	21 (20.6)	18 (17.6)	89.2	0.65
**Loaded reach**	Sighing	38 (37.3)	34 (33.3)	96.1	0.91
	Breath-holding	84 (82.4)	84 (82.4)	100.0	1.00
	Grimacing	28 (27.5)	24 (23.5)	86.3	0.64
	Guarding	78 (76.5)	78 (76.5)	100.0	1.00
	Rubbing	5 (4.9)	0 (0.0)	95.1	0.00
**50-foot walk**	Sighing	23 (22.5)	19 (18.6)	92.2	0.76
	Breath-holding	13 (12.7)	13 (12.7)	96.1	0.82
	Grimacing	5 (4.9)	8 (7.8)	93.1	0.43
	Guarding	12 (11.8)	3 (2.9)	87.3	0.09
	Rubbing	5 (4.9)	3 (2.9)	96.1	0.48
	Antalgic gait	23 (22.5)	16 (15.7)	93.1	0.78

Agreement was calculated for the presence or absence of each pain behavior during the first testing session. Antalgic gait was evaluated only during walking-related tasks. NC indicates that kappa could not be calculated because both raters recorded no occurrences of the behavior. R1, Rater 1; R2, Rater 2; κ, kappa coefficient.

**Table 5:** Correlations between Turkish PaBS scores and clinical and physical performance measures.

Measure	Spearman’s ρ	*p* Value
**Self-report measures**		
SF-MPQ affective score	0.82	<0.001
SF-MPQ sensory score	0.84	<0.001
SF-MPQ total score	0.85	<0.001
MPQ-VAS pain intensity	0.86	<0.001
ODI disability, %	0.85	<0.001
FABQ physical activity	0.93	<0.001
FABQ work	0.84	<0.001
FABQ total score	0.90	<0.001
**Physical performance measures**		
Repeated trunk flexion, s	0.93	<0.001
Repeated sit-to-stand, s	0.93	<0.001
Timed Up and Go, s	0.93	<0.001
Loaded reach, cm	−0.93	<0.001
50-foot walk, s	0.67	<0.001

Correlations were calculated using the first-session PaBS total score rated by Rater 1. Positive correlations were expected for pain, disability, fear-avoidance beliefs, and time-based performance measures; a negative correlation was expected for loaded reach distance. PaBS, Pain Behaviour Scale; SF-MPQ, Short-Form McGill Pain Questionnaire; MPQ-VAS, McGill Pain Questionnaire Visual Analog Scale; ODI, Oswestry Disability Index; FABQ, Fear-Avoidance Beliefs Questionnaire.

**Table 6:** Bland–Altman Agreement for PaBS Total Scores.

Comparison	Mean Difference	SD Difference	95% Limits of Agreement
Interrater agreement: R2 − R1, first session	−0.03	1.05	−2.08 to 2.02
Intrarater agreement: second − first session, R1	−0.74	0.94	−2.58 to 1.11

Bland–Altman analyses were performed using PaBS total scores. Interrater differences were calculated as Rater 2 minus Rater 1 during the first testing session. Intrarater differences were calculated as the second session minus the first session for Rater 1. PaBS, Pain Behaviour Scale; R1, Rater 1; R2, Rater 2.

## Data Availability

The datasets generated and/or analyzed during the current study are available from the corresponding author upon reasonable request.

## Abbreviations

BMI	Body Mass Index
CI	Confidence Interval
COSMIN	COnsensus-based Standards for the selection of health Measurement INstruments
FABQ	Fear-Avoidance Beliefs Questionnaire
GRRAS	Guidelines for Reporting Reliability and Agreement Studies
ICC	Intraclass Correlation Coefficient
MDC	Minimal Detectable Change
MDC95	Minimal Detectable Change at the 95% Confidence Level
MPQ-VAS	McGill Pain Questionnaire Visual Analog Scale
NC	Not Calculable
NSLBP	Nonspecific Low Back Pain
ODI	Oswestry Disability Index
PaBS	Pain Behaviour Scale
R1	Rater 1
R2	Rater 2
SD	Standard Deviation
SEM	Standard Error of Measurement
SF-MPQ	Short-Form McGill Pain Questionnaire
SPSS	Statistical Package for the Social Sciences
TUG	Timed Up and Go
